# Citation analytics: Data exploration and comparative analyses of *CiteScores* of Open Access and Subscription-Based publications indexed in *Scopus* (2014–2016)

**DOI:** 10.1016/j.dib.2018.05.005

**Published:** 2018-05-09

**Authors:** Aderemi A. Atayero, Segun I. Popoola, Jesse Egeonu, Olumuyiwa Oludayo

**Affiliations:** a Department of Electrical and Information Engineering, Covenant University, Ota, Nigeria; b Department of Economics and Development Studies, Covenant University, Ota, Nigeria; c Department of Business Management, Covenant University, Ota, Nigeria

**Keywords:** Citation impact, Open Access, CiteScore, Analytics, Citation analytics, Smart campus, Data mining

## Abstract

Citation is one of the important metrics that are used in measuring the relevance and the impact of research publications. The potentials of citation analytics may be exploited to understand the gains of publishing scholarly peer-reviewed research outputs in either Open Access (OA) sources or Subscription-Based (SB) sources in the bid to increase citation impact. However, relevant data required for such comparative analysis must be freely accessible for evidence-based findings and conclusions. In this data article, citation scores (*CiteScores*) of 2542 OA sources and 15,040 SB sources indexed in *Scopus* from 2014 to 2016 were presented and analyzed based on a set of five inclusion criteria. A robust dataset, which contains the *CiteScores* of OA and SB publication sources included, is attached as supplementary material to this data article to facilitate further reuse. Descriptive statistics and frequency distributions of OA *CiteScores* and SB *CiteScores* are presented in tables. Boxplot representations and scatter plots are provided to show the statistical distributions of OA *CiteScores* and SB *CiteScores* across the three sub-categories (Book Series, Journal, and Trade Journal). Correlation coefficient and p-value matrices are made available within the data article. In addition, Probability Density Functions (PDFs) and Cumulative Distribution Functions (CDFs) of OA *CiteScores* and SB *CiteScores* are computed and the results are presented using tables and graphs. Furthermore, Analysis of Variance (ANOVA) and multiple comparison post-hoc tests are conducted to understand the statistical difference (and its significance, if any) in the citation impact of OA publication sources and SB publication source based on *CiteScore*. In the long run, the data provided in this article will help policy makers and researchers in Higher Education Institutions (HEIs) to identify the appropriate publication source type and category for dissemination of scholarly research findings with maximum citation impact.

**Specifications Table**

**Table:** 

Subject area	*Data Analytics*
More specific subject area	*Citation Analytics*
Type of data	*Tables, graphs, figures, and spreadsheet file*
How data was acquired	*Data was acquired from publication source list available in Scopus online database**[1]**. A set of five inclusion criteria was established namely: publication source must be indexed in the Scopus database; publication source must be active as at 28th December 2017; publication must be written in English language; publication source type must either be Book Series, Journal or Trade Journal; and publication source must have CiteScores in 2014, 2015, and 2016.*
Data format	*Secondary, analyzed*
Experimental factors	*Publication sources that did not meet any of the five criteria for inclusion in the period under consideration were excluded.*
Experimental features	*Descriptive statistics, boxplot representations, scatter plots, frequency distributions, correlation and regression analyses, Probability Density Functions (PDFs), Cumulative Distribution Functions (CDFs), Analysis of Variance (ANOVA) test, and multiple post-hoc test are performed to explore the dataset provided in this data article. All statistical computations were done using the Machine Learning and Statistics toolbox in MATLAB 2016a software.*
Data source location	*Data is available as supplementary material to this data article*
Data accessibility	*In a bid to facilitate further works on citation analytics, detailed datasets are made publicly available in a Microsoft Excel spreadsheet file.*

**Value of the data**

• The dataset generated and made publicly available based on the stipulated criteria will help foster further investigation into the importance of *Elsevier CiteScore* and other source ranking methods [2], [3], [4].
• Presenting this data in open access format will help researchers identify relevant sources as veritable outlets for dissemination of their research findings [5], [6].
• Quite a lot of research findings often end up in subscription-only sources. This invariably limits access to such works and reduces their impact on future research significantly. This shortfall is mitigated by isolating and analyzing the OA sources of the largest global indexing body for scientific research [7], [8], [9].
• Descriptive statistics, frequency distributions, one-way ANOVA and multiple comparison post-hoc tests that are presented in tables, plots, and graphs will make data interpretation much easier for useful insights, inferences, and logical conclusions [10], [11], [12], [13].
• Detailed datasets that are made publicly available in a Microsoft Excel spreadsheet file attached to this article will encourage further explorative studies in this field of research.

## 1 Data

Analytics seeks to discover, interpret, and effectively communicate patterns in any given dataset. These attributes explain why analytics is becoming pervasive across various disciplines including ranking of Higher Education Institutions (HEIs). A very high premium is placed on scholarly research output as evidenced by publication in relevant sources as a proxy measure of excellence in ranking of HEIs. *Scopus* by Elsevier is currently the world's largest abstract and citation database of peer-reviewed literature. It currently boasts over 70 million records. *CiteScore™*– a measure of the average citations received per document published in a serial, is one of the three major indices used by *Scopus* to rank publication sources [14], [15], [16]. In this source ranking method, higher is better. This metric invention from *Scopus* is comprehensive and transparent. It is a free metrics of current sources indexed in *Scopus*.

The potentials of citation analytics may be exploited to understand the gains of publishing scholarly peer-reviewed research outputs in either Open Access (OA) sources or Subscription-Based (SB) sources in the bid to increase citation impact. However, relevant data required for such comparative analysis must be freely accessible for evidence-based findings and conclusions. In this data article, citation scores (*CiteScores*) of 2542 OA sources and 15,040 SB sources indexed in *Scopus* from 2014 to 2016 were presented and analyzed based on a set of five inclusion criteria. Two publication source types (OA and SB) and they both covered three sub-categories namely*: Book Series*; *Journal*; and *Trade Journal*. Precise information about the distribution of the *CiteScore* data across the source types and sub-categories is presented in Table 1. Under the OA source type, 5 Book Series sources, 2536 Journal sources, and 1 Trade Journal source successfully met the inclusion criteria. On the other hand, 378 Book Series sources, 14,448 Journal sources, and 214 Trade Journal sources were included under the SB source type based on the inclusion criteria that were earlier set. It is becoming increasingly popular for subscription-based source providers to grant authors right to open their articles for a fee. This practice is sometimes referred to as the hybrid model. However, we noted that the hybrid model is a subset of the subscription-based model. Hence, in this data article, the hybrid model is totally captured under the SB category.

**Table 1:** Classification of scholarly research output publications.

	Open Access (OA)	Subscription (SB)	Total
Book Series	5	378	383
Journal	2536	14,448	16,984
Trade Journal	1	214	215
Total	2542	15,040	

## 2 Experimental design, materials and methods

In this data article, *CiteScores* of 2542 OA sources and 15,040 SB sources indexed in *Scopus* from 2014 to 2016 were presented and analyzed. The methodology for calculating the CiteScore metrics is quite easy as represented by Eqs. (1), (2). The methodology is further explained and illustrated in Fig. 6. CiteScore for year *N* (CiteScore *N*) sums the citations received in year *N* to documents published in years *N-1, N-2, and N-3*, and divides this by the number of documents published in the three consecutive years *N-1, N-2, and N-3.*

(1) $$\mathrm{CiteScore}\;N\;=\;\frac{\mathrm{Citation}\;\mathrm{Count}\;\mathrm{in}\;N}{\mathrm{Documents}\;\left(N-3\right)-\left(N-1\right)}$$

For instance,

(2) $$\mathrm{CiteScore}\;2016\;=\;\frac{\mathrm{Citation}\;\mathrm{Count}\;\mathrm{in}\;\;2016}{\mathrm{Documents}\;2013-2015}$$

According to *Scopus*, the 3-year CiteScore time window was chosen as a best fit for all subject areas. Research shows that a 3-year publication window is long enough to capture the citation peak of the majority of disciplines. A set of five inclusion criteria was established namely: publication source must be indexed in the *Scopus* database; publication source must be active as at 28th December 2017; publication must be written in English language; publication source type must either be Book Series, Journal or Trade Journal; and publication source must have *CiteScores* in 2014, 2015, and 2016. The Source identification numbers were carefully anonymized using the format: OA##### for OA publication sources and; SB##### for SB publication sources, where *#* is an integer. Hence, the sequential Publication ID is OA00001 through OA2542 for OA publication sources, and SB00001 through SB15040 for SB publication sources.

**Fig. 1:**
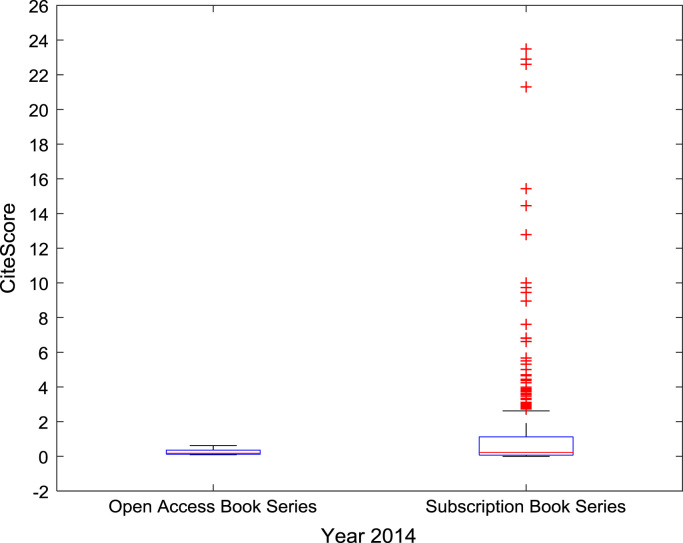
Boxplot representation of *CiteScore* data of Book Series sources in 2014.

**Fig. 2:**
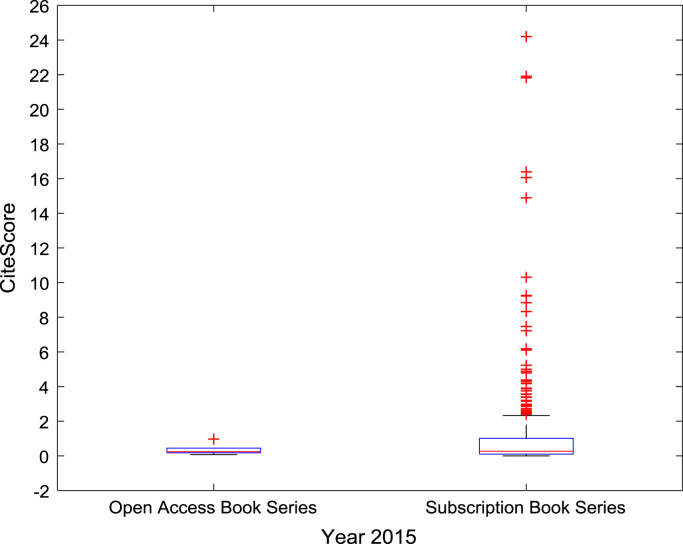
Boxplot representation of *CiteScore* data of Book Series sources in 2015.

**Fig. 3:**
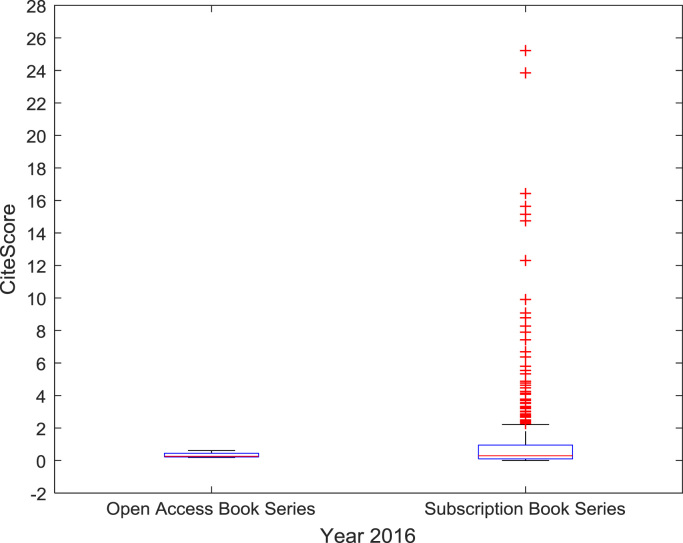
Boxplot representation of *CiteScore* data of Book Series sources in 2016.

**Fig. 4:**
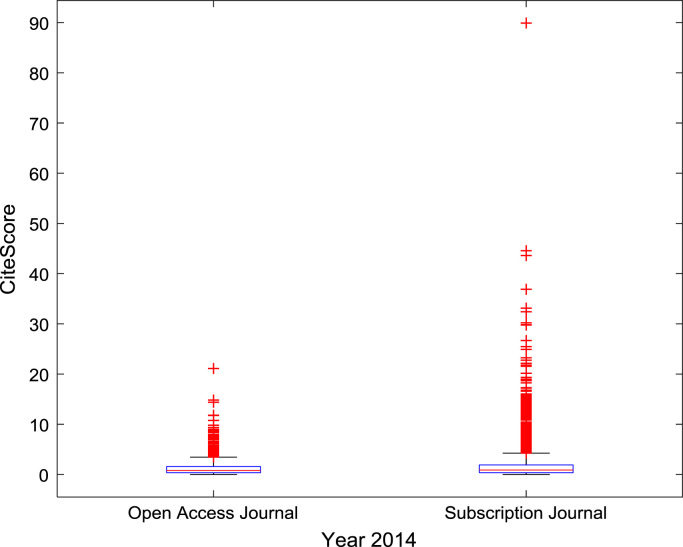
Boxplot representation of *CiteScore* data of Journal sources in 2014.

**Fig. 5:**
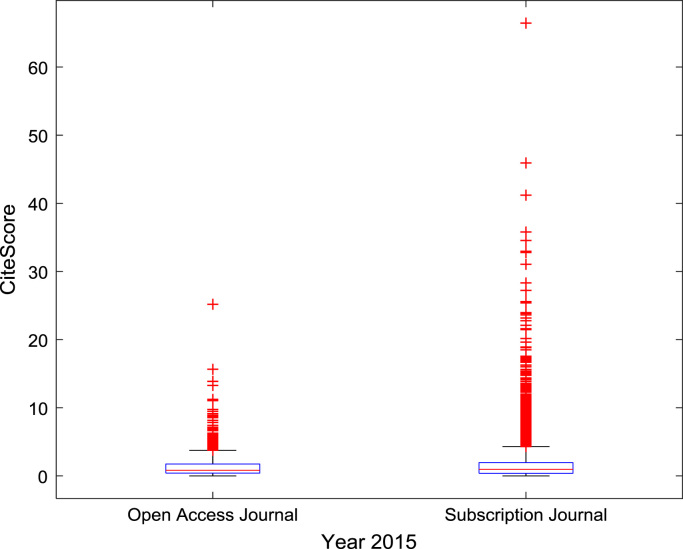
Boxplot representation of *CiteScore* data of Journal sources in 2015.

**Fig. 6:**
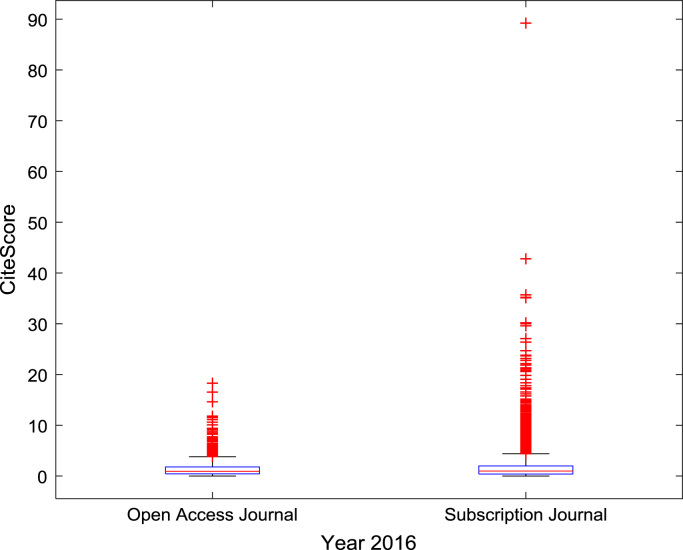
Boxplot representation of *CiteScore* data of Journal sources in 2016.

**Fig. 7:**
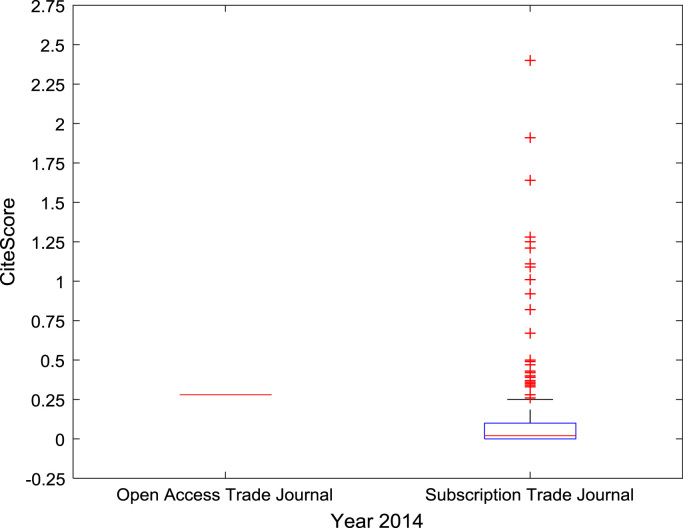
Boxplot representation of *CiteScore* data of Trade Journal sources in 2014.

**Fig. 8:**
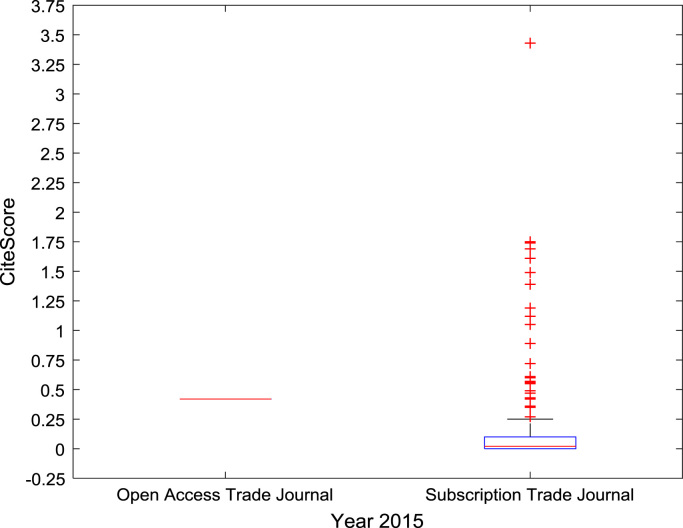
Boxplot representation of *CiteScore* data of Trade Journal sources in 2015.

**Fig. 9:**
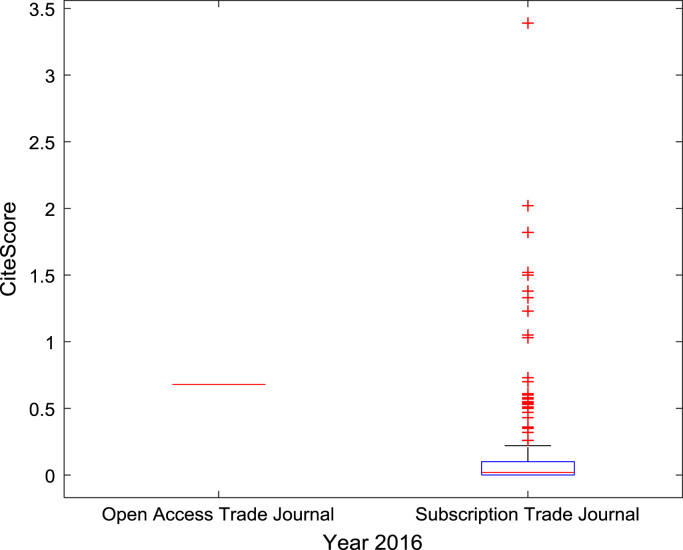
Boxplot representation of *CiteScore* data of Trade Journal sources in 2016.

The descriptive statistics of the *CiteScores* of OA and SB scholarly research output sources for the three-year period are as presented in Table 2. In order to measure the tendency of centrality in the *CiteScore* data, boxplots are drawn for each publication source type. The boxplot representations of *CiteScore* data of Book Series, Journal, and Trade Journal sources for 2014, 2015, and 2016 are shown in Fig. 1, Fig. 2, Fig. 3, Fig. 4, Fig. 5, Fig. 6, Fig. 7, Fig. 8, Fig. 9.

**Table 2:** Descriptive statistics of *CiteScore* data of scholarly research outputs (2014–2016).

	2014		2015		2016	
	*Open Access (OA)*	*Subscription (SB)*	*Open Access (OA)*	*Subscription (SB)*	*Open Access (OA)*	*Subscription (SB)*
Mean	1.22	1.42	1.32	1.47	1.37	1.50
Median	0.78	0.85	0.82	0.92	0.92	0.94
Mode	0.00	0.00	0.00	0.00	0.12	0.00
Standard Deviation	1.41	2.13	1.51	2.09	1.49	2.14
Variance	1.98	4.55	2.29	4.38	2.23	4.58
Kurtosis	31.72	256.26	39.57	127.03	23.11	240.01
Skewness	3.77	9.84	4.10	7.51	3.31	9.41
Range	21.11	89.91	25.19	66.45	18.29	89.23
Minimum	0.00	0.00	0.00	0.00	0.00	0.00
Maximum	21.11	89.91	25.19	66.45	18.29	89.23
Total Samples	2542	15,040	2542	15,040	2542	15,040

**Fig. 10:**
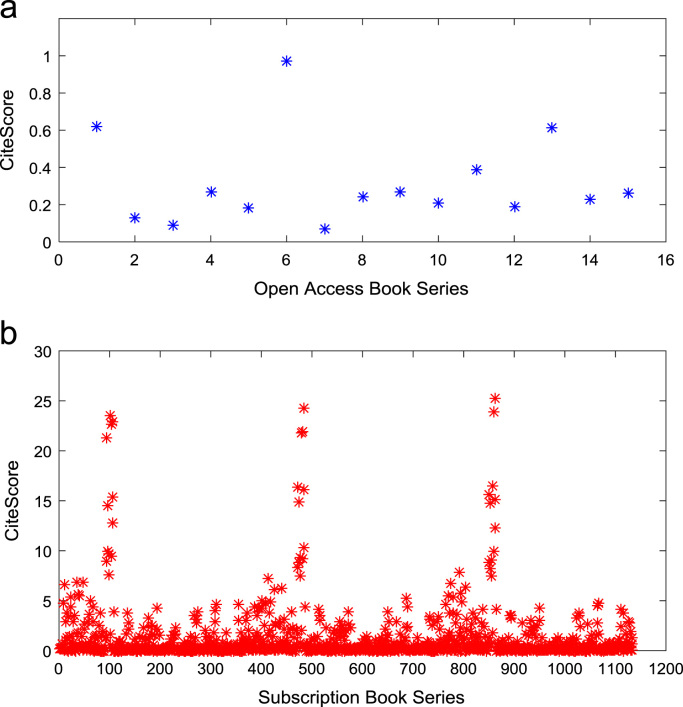
Scatter plot of (a) OA (b) SB Book Series *CiteScore* data (2014–2016).

**Fig. 11:**
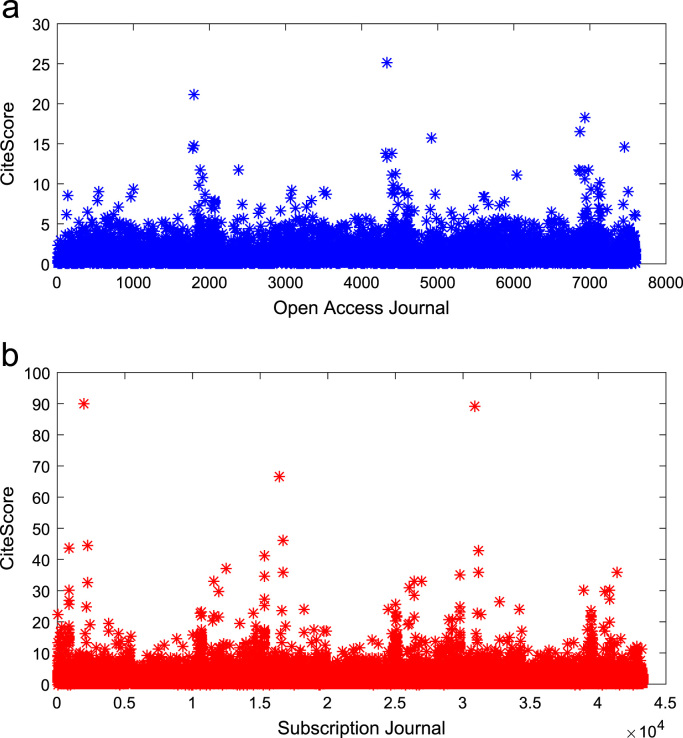
Scatter plot of (a) OA (b) SB Journal *CiteScore* data (2014–2016).

**Fig. 12:**
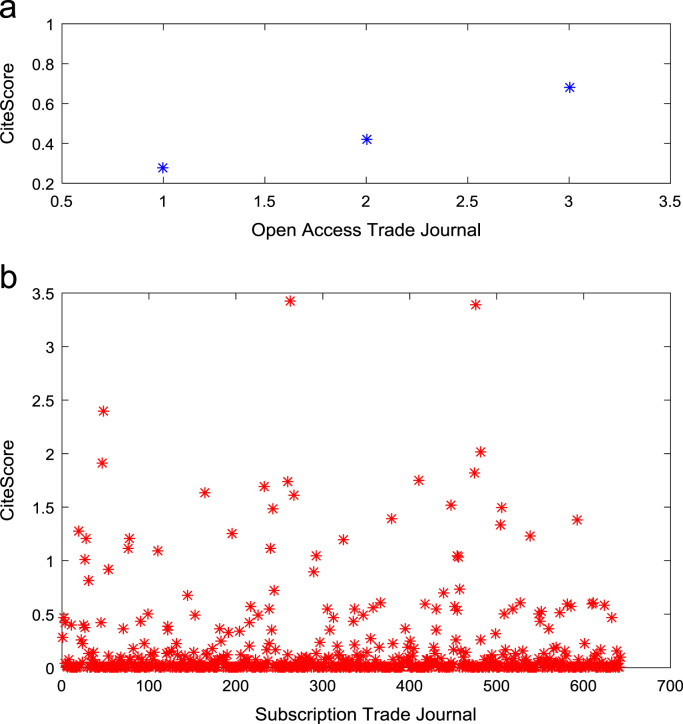
Scatter plot of (a) OA (b) SB Trade Journal *CiteScore* data (2014–2016).

**Fig. 13:**
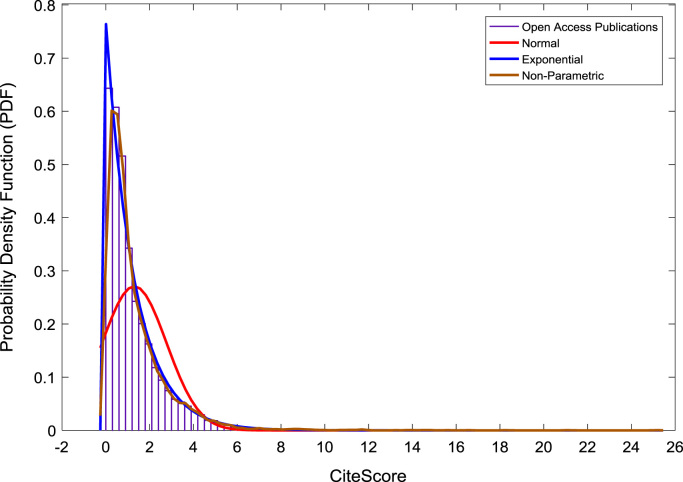
Probability density function plot of OA publications.

**Fig. 14:**
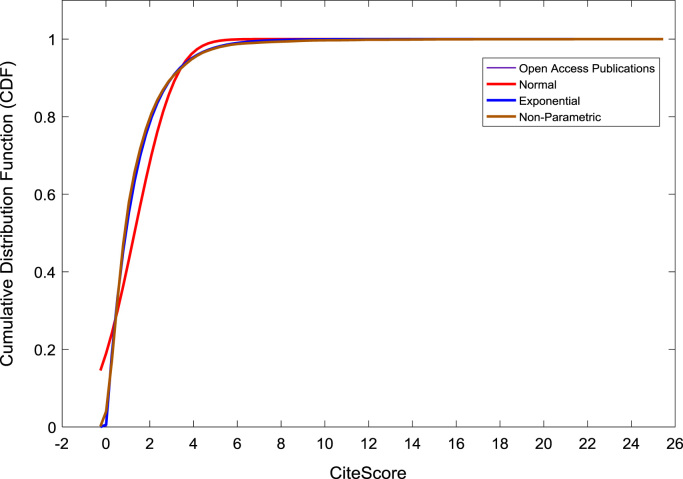
Cumulative distribution function plot of OA publications.

**Fig. 15:**
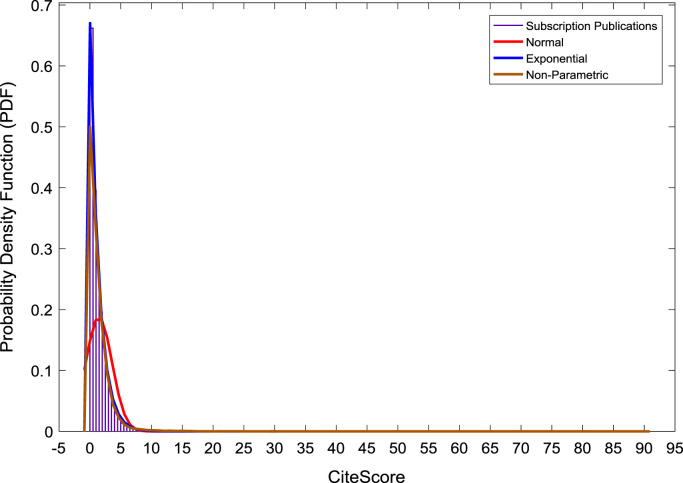
Probability density function plot of SB publications.

**Fig. 16:**
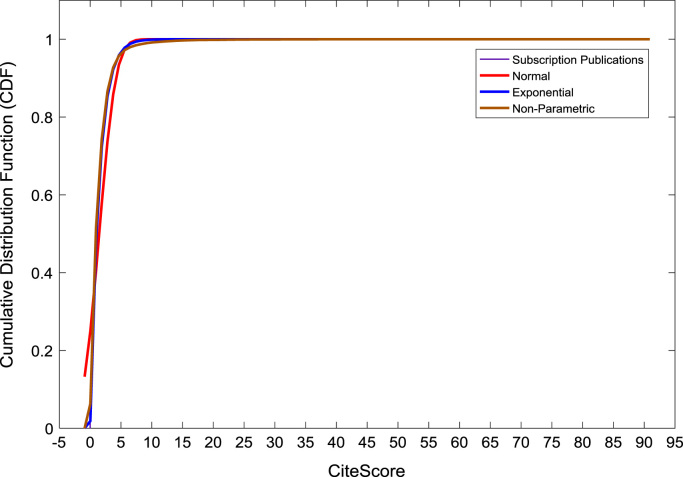
Cumulative distribution function plot of SB publications.

Fig. 10, Fig. 11, Fig. 12 show the trends in the *CiteScores* of OA and SB publication sources in the sub-categories of Book Series, Journal, and Trade Journal respectively between 2014 and 2016. Probability Density Functions (PDFs) and Cumulative Distribution Functions (CDFs) of the dataset are also computed. PDF and CDF models of Normal, Exponential, and Non-parametric distributions were used to fit the OA and SB *CiteScore* data and the results are shown in Fig. 13, Fig. 14, Fig. 15, Fig. 16 respectively. Distribution fitting parameters for OA *CiteScore* data, and their estimates and standard errors, are presented in Tables 3 and 4 respectively. In like manner, the distribution fitting parameters for SB *CiteScore* data, and their estimates and standard errors, are presented in Tables 5 and 6 respectively.

**Table 3:** Distribution fitting parameters for OA *CiteScore* data (2014–2016).

	Normal	Exponential
Log Likelihood	−13770.7	−9634.67
Domain	−∞<*y*<∞	0<*y*<∞
Mean	1.3013	1.3013
Variance	1.4724	1.6935

**Table 4:** Estimates and standard errors for OA *CiteScore* data distribution (2014–2016).

	Normal		Exponential	
Parameter	Approx	Std Err	Approx	Std Err
*µ*	1.3013	0.0169	1.3013	0.0149
*σ*	1.4724	0.0119	–	–

**Table 5:** Distribution fitting parameters for SB *CiteScore* data (2014–2016).

	Normal	Exponential
Log Likelihood	–13770.7	−9634.67
Domain	−∞ <*y*<∞	0<*y*<∞
Mean	1.3013	1.3013
Variance	1.4724	1.6935

**Table 6:** Estimates and standard errors for OA *CiteScore* data distribution (2014–2016).

	Normal		Exponential	
Parameter	Approx	Std Err	Approx	Std Err

*µ*	1.3013	0.0169	1.3013	0.0149
*σ*	1.4724	0.0119	–	–

**Fig. 17:**
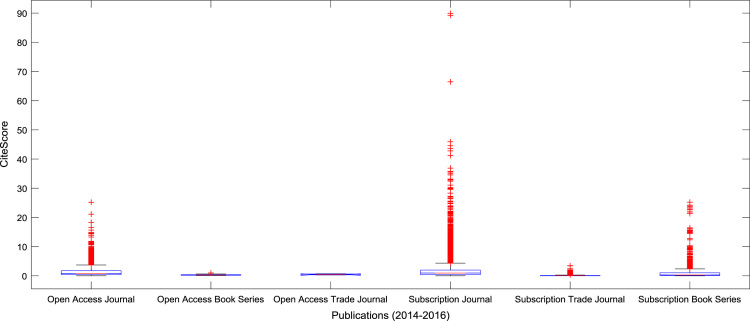
Boxplot showing the comparison of *CiteScores* of publication sources.

**Fig. 18:**
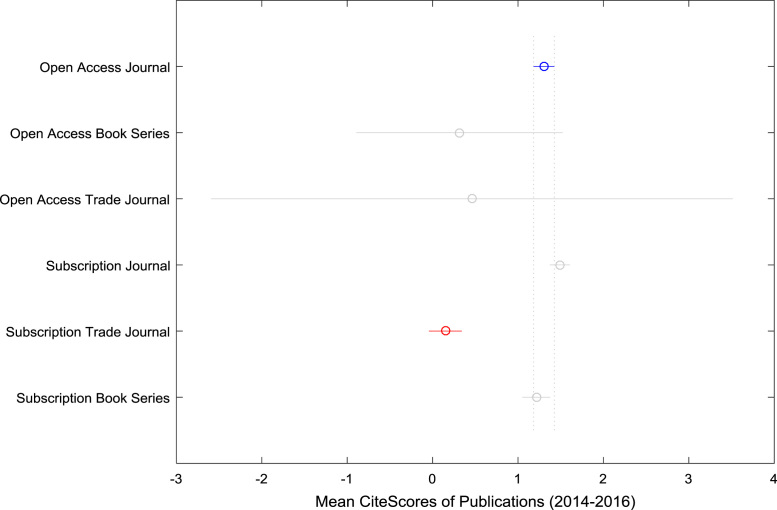
Multiple comparison post-hoc plot of *CiteScore* data (2014–2016).

Furthermore, correlation analyses are performed to establish a linear relationship between the OA *CiteScores* and the SB *CiteScores*. The correlation coefficient matrices and their corresponding *p*-values are presented in Table 7, Table 8, Table 9, Table 10, Table 11, Table 12. Analysis of Variance (ANOVA) and multiple comparison post-hoc tests are conducted to understand the statistical difference (and its significance, if any) in the citation impact of OA publication sources and SB publication source based on *CiteScore*. The results of the ANOVA test and the multiple comparison post-hoc test are presented in Tables 13 and 14. The mean *CiteScores* of the six groups (Open Access Book Series, Open Access Journal, Open Access Trade Journal, Subscription Book Series, Subscription Journal, and Subscription Trade Journal) are shown in Figs. 17 and 18 to aid comparative analyses.

**Table 7:** Correlation coefficient matrix of Book Series *CiteScore* data (2014–2016).

		2014	2015	2016
Open Access Book Series	2014	1		
	2015	0.9566	1	
	2016	−0.0216	0.2624	1
Subscription Book Series	2014	1		
	2015	0.9828	1	
	2016	0.9696	0.9820	1

**Table 8:** *P*-value matrix of Book Series *CiteScore* data (2014–2016).

		2014	2015	2016
Open Access Book Series	2014	1		
	2015	0.0108	1	
	2016	0.9725	0.6698	1
Subscription Book Series	2014	1		
	2015	0.0000	1	
	2016	0.0000	0.0000	1

**Table 9:** Correlation coefficient matrix of Journal *CiteScore* data (2014–2016).

		2014	2015	2016
Open Access Journal	2014	1		
	2015	0.9549	1	
	2016	0.8986	0.9480	1
Subscription Journal	2014	1		
	2015	0.9780	1	
	2016	0.9668	0.9783	1

**Table 10:** *P*-value matrix of Journal *CiteScore* data (2014–2016).

		2014	2015	2016
Open Access Journal	2014	1		
	2015	0.0000	1	
	2016	0.0000	0.0000	1
Subscription Journal	2014	1		
	2015	0.0000	1	
	2016	0.0000	0.0000	1

**Table 11:** Correlation coefficient matrix of Trade Journal *CiteScore* data (2014–2016).

		2014	2015	2016
Open Access Trade Journal	2014	1		
	2015	1.0000	1	
	2016	1.0000	1.0000	1
Subscription Trade Journal	2014	1	0.9614	0.9320
	2015	0.9614	1	0.9405
	2016	0.9320	0.9405	1

**Table 12:** *P*-value matrix of Trade Journal *CiteScore* data (2014–2016).

		2014	2015	2016
Open Access Trade Journal	2014	1		
	2015	1.0000	1	
	2016	1.0000	1.0000	1
Subscription Trade Journal	2014	1	0.0000	0.0000
	2015	0.0000	1	0.0000
	2016	0.0000	0.0000	1

**Table 13:** ANOVA test results on *CiteScore* data (2014–2016).

Source of variation	Sum of squares	Degree of freedom	Mean squares	*F* statistic	*P*-value
Group (Between)	1401.3	5	280.268	67.66	9.79×10^–71^
Error (Within)	218460.7	52740	4.142		
Total	219862	52745

**Table 14:** Multiple comparison post-hoc test results.

Source type	Source type	Mean difference	Lower Limit (95% confidence intervals)	Upper Limit (95% confidence intervals)	*P*-value
Open Access Journal	Open Access Book Series	−0.5107	0.9883	2.4873	0.4152
Open Access Journal	Open Access Trade Journal	−2.5056	0.8436	4.1928	0.9799
Open Access Journal	Subscription Journal	−0.2590	−0.1869	−0.1148	0.0000
Open Access Journal	Subscription Trade Journal	0.9158	1.1542	1.3925	0.0000
Open Access Journal	Subscription Book Series	−0.0942	0.0904	0.2750	0.7302
Open Access Book Series	Open Access Trade Journal	−3.8128	−0.1447	3.5235	1.0000
Open Access Book Series	Subscription Journal	−2.6729	−1.1751	0.3226	0.2212
Open Access Book Series	Subscription Trade Journal	−1.3490	0.1659	1.6808	0.9996
Open Access Book Series	Subscription Book Series	−2.4053	−0.8979	0.6095	0.5334
Open Access Trade Journal	Subscription Journal	−4.3791	−1.0305	2.3182	0.9521
Open Access Trade Journal	Subscription Trade Journal	−3.0458	0.3105	3.6669	0.9998
Open Access Trade Journal	Subscription Book Series	–4.1062	−0.7532	2.5997	0.9880
Subscription Journal	Subscription Trade Journal	1.1104	1.3410	1.5716	0.0000
Subscription Journal	Subscription Book Series	0.1028	0.2772	0.4517	0.0001
Subscription Trade Journal	Subscription Book Series	−1.3503	−1.0638	−0.7773	0.0000
